# Screening for Psychiatric Comorbidities and Psychotherapeutic Assessment in Inpatient Epilepsy Care: Preliminary Results of an Implementation Study

**DOI:** 10.3389/fnint.2021.754613

**Published:** 2021-10-12

**Authors:** Rosa Michaelis, Sabine Schlömer, Anja Lindemann, Vanessa Behrens, Wenke Grönheit, Milena Pertz, Stephanie Rammé, Sabine Seidel, Tim Wehner, Jörg Wellmer, Uwe Schlegel, Stoyan Popkirov

**Affiliations:** ^1^Department of Neurology, University Hospital Knappschaftskrankenhaus Bochum, Ruhr University Bochum, Bochum, Germany; ^2^Faculty of Health, Witten/Herdecke University, Witten-Herdecke, Germany; ^3^Ruhr-Epileptology, Department of Neurology, University Hospital Knappschaftskrankenhaus Bochum, Ruhr University Bochum, Bochum, Germany

**Keywords:** depression, anxiety, psychogenic non-epileptic seizures, psychiatric comorbidities in epilepsy, psychological treatments

## Abstract

**Background:** Anxiety and depression remain underdiagnosed in routine clinical practice in up to two thirds of epilepsy patients despite significant impact on medical and psychosocial outcome. Barriers to adequate mental health care for epilepsy and/or psychogenic non-epileptic seizures (PNES) include a lack of integrated mental health specialists and standardized procedures. This naturalistic study outlines the procedures and outcome of a recently established psychotherapeutic service.

**Methods:** Routine screening included the Neurological Disorders Depression Inventory for Epilepsy (NDDI-E, cut-off value > 13) and Generalized Anxiety Disorder scale (GAD-7, cut-off value > 5). Positively (above cut-off in at least one questionnaire) screened patients were seen for a standardized interview for mental health disorders and the development of a personalized treatment plan. PNES patients were seen irrespective of their screening score. Resources were provided to support self-help and access to psychotherapy. Patients were contacted 1 month after discharge to evaluate adherence to therapeutic recommendations.

**Results:** 120 patients were screened. Overall, 56 of 77 positively screened patients (77%) were found to have a psychiatric diagnosis through standardized interview. More epilepsy patients with an anxiety disorder had previously been undiagnosed compared to those with a depressive episode (63% vs. 30%); 24 epilepsy patients (62%) with a psychiatric comorbidity and 10 PNES patients (59%) were not receiving any mental health care. At follow-up, 16/17 (94%) epilepsy patients and 7/7 PNES patients without prior psychiatric treatment were adhering to therapeutic recommendations.

**Conclusion:** Integrating mental health specialists and establishing standardized screening and follow-up procedures improve adherence to mental health care recommendations in epilepsy and PNES patients.

## Introduction

One in five patients with epilepsy has comorbid anxiety or depression (Mula et al., 2021). Psychiatric comorbidities have been associated with a poor response to medical treatment, increased morbidity and mortality, and adverse psychosocial outcome (Kanner et al., 2010; Petrovski et al., 2010). Thus, their early identification and treatment with pharmacological and/or psychological therapy is strongly recommended (Kerr et al., 2011; Michaelis et al., 2018). However, the literature suggests that anxiety and depression remain underdiagnosed and undertreated in routine clinical practice at a rate of 64 and 33%, respectively (Gandy et al., 2021; Scott et al., 2021).

Reflecting this, the inclusion of routine screening for anxiety and depression in patients with epilepsy has been included in the American Academy of Neurology’s quality indicators (Patel et al., 2018). Indeed, the use of an epilepsy-specific screening measure for depression, the Neurological Disorders Depression Inventory for Epilepsy (NDDI-E; Gilliam et al., 2006) has significantly improved the detection rate of depression in epilepsy patients in busy clinical practice settings (Friedman et al., 2009). In the area of anxiety, the seven-item Generalized Anxiety Disorder scale (GAD-7; Löwe et al., 2008) is commonly used as a non-specific screening measure for anxiety in patients with epilepsy (Seo et al., 2014).

Psychogenic non-epileptic seizures (PNES) are an important differential diagnosis and a common neuropsychiatric comorbidity of epilepsy (LaFrance et al., 2013). Long-term prognosis of PNES has been shown to be poor in patients with PNES after the communication of the diagnosis in an epilepsy monitoring unit setting (Walther et al., 2019). Psychological interventions have been recognized as the treatment of choice for PNES (Goldstein et al., 2020) and adherence to psychotherapy yields significant improvement in PNES frequency and quality of life (Tolchin et al., 2019). Thereby, determining psychiatric comorbidity is key to a personalized understanding and treatment of PNES (Popkirov et al., 2019).

The Psychology Task Force of the International League Against Epilepsy (ILAE) has recently published the findings of an online survey of epilepsy health professionals’ practices in managing psychiatric comorbidities in patients with epilepsy (Gandy et al., 2021). This study highlighted many barriers to mental health care for patients with epilepsy and/or PNES such as a lack of both trained mental health specialists and standardized procedures in epilepsy care settings. Indeed, a majority of epilepsy professionals felt that resources should be provided that guide the integration of psychological care within epilepsy care, especially in light of the World Health Assembly 2020 call for urgent resolution of mental health issues in epilepsy and other neurological disorders.

In this naturalistic study we aim at outlining characteristics of a recently established psychotherapeutic service in an inpatient epilepsy care setting as well as the benefit and adoption of therapeutic recommendations that were observed in this context.

## Methods

This study was conducted at the Ruhr-Epileptology, a tertiary epilepsy center nested within the Neurology Department of the University Hospital Knappschaftskrankenhaus in Bochum, Germany. We report the interim results of participants who were seen between January 2021 and June 2021 within a newly established psychotherapy service.

The main aims of this study were to identify:

1. **Diagnostic yield:** How many patients received a new psychiatric diagnosis?

2. **Adoption of treatment recommendations:** How many patients with a psychiatric diagnosis without prior mental health care reported adherence to treatment recommendations at 1 month follow-up after discharge?

### Ethical Aspects

Ethical approval was obtained from the ethics board of the Ruhr University Bochum (20-7127). All patients gave written informed consent to participate in the study and for anonymized data to be included in publications.

### Participants

We consecutively recruited German-speaking adults (≥18 years) with an admission appointment in the Ruhr-Epileptology. Exclusion criteria comprised clinically relevant barriers to psychotherapeutic sessions, such as language barriers, acute psychiatric disorders that warrant primarily pharmacological treatment (e.g., alcohol withdrawal, delirium), notable severe cognitive impairment (e.g., in patients with developmental delay due to infantile brain damage), or evident acute psychiatric disorders that warrant immediate referral to psychiatry (e.g., psychosis, suicidality) established during the neurological admission examination or history taking.

### Screening Procedures

Routine screening for psychiatric comorbidities at the Ruhr-Epileptology included the systematic completion of the paper-based NDDI-E (sensitivity of 83.7% at the used cut-off value of > 13; Gill et al., 2017) and GAD-7 (sensitivity of 88.1% at the used cut-off value of > 5; Seo et al., 2014) questionnaires by all inpatients with an admission appointment. The questionnaires were distributed and collected by an epilepsy specialist assistant (AL) (Pfäfflin et al., 2016) on the first or second day of the patients’ hospital stay.

### Psychotherapeutic Sessions

All patients whose scores exceeded a cut-off value (GAD-7 > 5 and/or NDDI-E > 13) were seen by a licensed psychotherapist (RM, SSch) for one session that included a freely available standardized interview for mental health disorders aiming at diagnostic evaluation of lifetime and current symptoms of anxiety disorders (including panic disorder, agoraphobia, social anxiety disorder, specific phobia, and generalized anxiety disorder), affective disorders (including unipolar and bipolar affective disorders), adjustment disorder, posttraumatic stress disorder, eating disorders, obsessive compulsive disorder, somatization disorder, psychotic symptoms, and suicidality (Margraf et al., 2017; Margraf, 2017) as well as the formulation and communication of a personalized psychological treatment plan. Patients with probable PNES were seen irrespective of their questionnaire scores. Sessions with patients with probable PNES focused on the development of a personalized seizure model as well as patient-oriented counseling aiming at the enhancement of motivation to behavioral change to address seizure triggers, warning signs and detrimental consequences of PNES (e.g., avoidance behavior). Some sessions included a short guided mindfulness-based exercise.

### Supportive Resources

Guided mindfulness-based exercises were recorded and sent to the patients. All patients who expressed interest in self-help materials and/or psychotherapeutic treatment were provided with personalized practical tips to bridge the waiting time as well as corresponding links to websites to support the systematic search for self-help books, self-help courses and/or therapists. The resources that were shared with the patients were personalized according to the patients’ diagnoses, preferences and interests.

### Follow-Up

The therapists (RM, SSch) asked permission to contact the patients through e-mail and/or phone 1 month after discharge to inquire about the development of symptoms and to discuss potential challenges concerning adherence to therapeutic recommendations.

### Data Analysis

Epilepsy details, previous diagnoses of depression, anxiety and/or PNES, current psychotropic medication and non-pharmacological treatment (e.g., psychotherapy) were retrieved from medical records. The patients’ clinical features including psychiatric diagnoses, current treatment and adherence to treatment recommendations at 1 month follow-up after discharge are summarized using descriptive statistical methods.

## Results

Between January 2021 and June 2021 120 patients were screened using the NDDI-E and the GAD-7. The scores of the NDDI-E and/or GAD-7 exceeded the cut-off values in 90/120 patients (75%). Fifty-eight of these 90 patients (64%) were discharged with a diagnosis of epilepsy according to the criteria of the ILAE, 16 with PNES (18%) and 16 with non-epileptic events other than PNES (18%).

Seven of the 58 epilepsy patients (12%) were excluded due to a language barrier or cognitive impairment and six patients (13%) were discharged prior to a psychotherapeutic session. A new psychiatric diagnosis was established in 22 of the 45 (49%) positively screened epilepsy patients who were seen by a psychotherapist, 16 of whom (73%) did not have a previous psychiatric diagnosis.

Overall, 18 of 90 positively screened patients (20%; 9 epilepsy patients and 9 patients with non-epileptic events other than PNES) were not found to have a psychiatric diagnosis through standardized interview, in line with the reported specificity of 86.3% (NDDI-E) and 87.9% (GAD-7) of the screening instruments (Gill et al., 2017; Wang et al., 2019).

**FIGURE 1:**
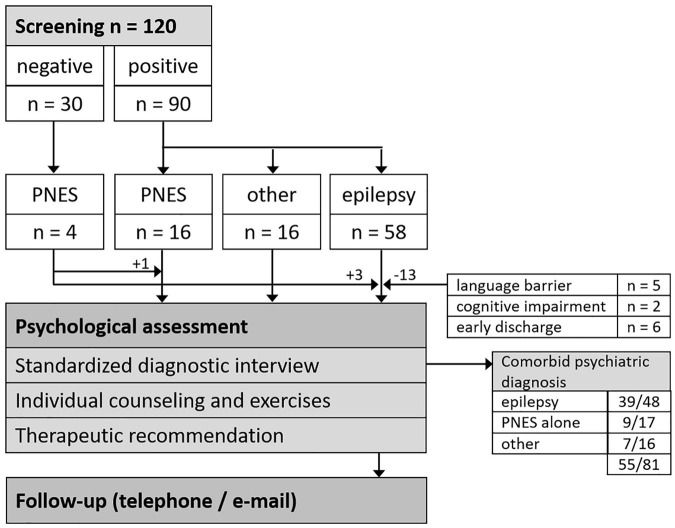
Patient flow chart. PNES, psychogenic non-epileptic seizures.

Four patients whose questionnaires did not exceed a cut-off value were seen due to comorbid PNES (*n* = 3) or PNES alone (*n* = 1). Altogether, 81 patients were seen for at least one psychotherapeutic session (see Figure 1).

### Psychiatric Comorbidities in Patients With Epilepsy

Overall, 39 epilepsy patients had a comorbid psychiatric diagnosis (see Figure 1; see Table 1 for demographic and medical characteristics). While the diagnosis of a depressive episode had previously been established in 16 of 23 epilepsy patients (70%) with a current depressive episode, only six of 16 epilepsy patients (37%) with an anxiety disorder had previously been diagnosed (see Table 2). Nine epilepsy patients (23%) were diagnosed with both a depressive episode and an anxiety disorder. Additional psychiatric comorbidities included adjustment disorder (*n* = 9), comorbid PNES (*n* = 7), bipolar affective disorder (*n* = 3), posttraumatic stress disorder (*n* = 2), borderline personality disorder (*n* = 2), eating disorder (*n* = 1), obsessive compulsive disorder (*n* = 1), and somatization disorder (*n* = 1).

**TABLE 1:** Epilepsy characteristics of epilepsy patients with psychiatric comorbidity.

Patient characteristics			Total *n* = 39	
Gender				
Female:male (%)			28:11	(72%:28%)
Age in years				
median (range)			39	(19–67)
**Epilepsy diagnosis**				
Total (%)				
Focal epilepsy			35	(90%)
	Structural		20	
		Hippocampal sclerosis	10	
		Malformation	5	
		Tumor	2	
		Vascular malformation	1	
		Hypoxic-ischemic	1	
		Porencephalic cyst	1	
	Unknown		12	
	Antibody-mediated		3	
Generalized epilepsy			4	(10%)
	Primary generalized		3	
	JME		1	
Age at onset in years				
median (range)			15	(2–59)
Epilepsy duration in years				
median (range)			19	(0.3–40)
**ASDs**				
Total (%)				
None			2	(5%)
1 ASD			18	(46%)
2 ASDs			7	(18%)
3 ASDs			12	(31%)
Pharmacoresistant			31	(80%)
	ASD monotherapy (*n* = 18)			
		Lamotrigine	9	
		Lacosamide	4	
		Levetiracetam	2	
		Brivaracetam	1	
		Carbamazepine	1	
		Valproic Acid	1	
ASD polytherapy (*n* = 19)				
		Lamotrigine	11	
		Levetiracetam	8	
		Lacosamide	7	
		Brivaracetam	6	
		Valproic Acid	6	
		Perampanel	5	
		Carbamazepine	1	
		Eslicarbazepine	1	
		Felbamate	1	
		Oxcarbazepine	1	
		Phenytoine	1	
		Pregabaline	1	
		Topiramate	1	

*ASD, Anti-seizure drug; JME, Juvenile Myoclonic Epilepsy.*

**TABLE 2:** Psychiatric comorbidities and treatment characteristics of epilepsy patients with psychiatric comorbidity.

Patient comorbidities and treatment characteristics				
**Comorbidities**			**Newly diagnosed**	
Total (%)			Total (%)	
Comorbid PNES	7	(18%)	1/7	(14%)
Adjustment disorder	9	(23%)	8/9	(89%)
Any depressive episode	23	(59%)	7/23	(30%)
Mild depressive episode	4	(10%)		
Moderate depressive episode	19	(49%)		
Any anxiety disorder	16	(41%)	10/16	(63%)
**Treatment**			**New treatment recommendation**	
Total (%)			Total (%)	
Psychotropic pharmacotherapy	7	(18%)	2/27	(7%)
Non-pharmacological treatment	3	(8%)	13/31	(42%)
Psychotropic pharmacotherapy and				
Non-pharmacological treatment	5	(13%)	4/34	(12%)

*PNES, Psychogenic non-epileptic seizures.*

Twelve epilepsy patients (31%) were currently taking antidepressant pharmacotherapy; five of these patients received additional psychotherapy; three epilepsy patients (8%) received non-pharmacological treatment (i.e., psychotherapy, psychiatric follow-up sessions) alone (see Table 2). Altogether, 24 epilepsy patients (62%) with a psychiatric comorbidity were not receiving any mental health care at the time of admission; in four of these 24 epilepsy patients (17%) a psychiatric diagnosis had previously been established.

#### Treatment Recommendations

Considering patients’ preferences, new treatment recommendations were discussed with 22 epilepsy patients (56%). Three of those 24 epilepsy patients (13%) who were not receiving any mental health care were not open to the discussion of treatment recommendations. Both pharmacotherapy and psychotherapy were recommended to four additional epilepsy patients (see Table 2). Self-help strategies alone were recommended to three epilepsy patients with mild disorders including adjustment disorder. Twenty of the 21 epilepsy patients (95%) who had received a treatment recommendation and who were not already receiving any treatment agreed to follow-up through phone and/or e-mail after discharge.

#### Follow-Up

Seventeen of the 20 epilepsy patients (85%) who had agreed to follow-up evaluation could be reached through phone or e-mail; 16 of these 17 epilepsy patients (94%) were adhering to therapeutic recommendations, i.e., 12 patients had requested to be put on a psychotherapist’s waiting list, four patients had started a psychotropic pharmacotherapy, and two patients were practicing self-help strategies.

### Patients With Psychogenic Non-epileptic Seizures Alone

Seventeen patients with PNES alone attended a psychotherapeutic session; 12 of these patients (71%) were women, their median age was 31 years (range: 18–56 years). In nine of these patients (53%) PNES had not previously been diagnosed. The median time interval since the first seizure was 1 year (range: < 1–35 years) in these newly diagnosed patients and 6 years (range: < 1–30 years) in the other eight patients with a previous diagnosis of PNES. The diagnostic procedures had included a suggestive seizure induction in three patients who had not shown a typical event during monitoring; in two patients a typical seizure could be recorded during the suggestive seizure induction which confirmed a certain diagnosis of PNES (Popkirov et al., 2020). Ten patients (59%) had previously been treated with Anti-Seizure Drugs (ASDs); nine of these PNES patients were still taking at least one ASD upon admission. ASDs were completely tapered in five of these patients during their hospital stay and a continuing outpatient ASD taper was recommended in all remaining patients. Nine PNES patients (53%) had previously been diagnosed with additional other psychiatric diagnoses including depression (*n* = 4), personality disorder (*n* = 3), and posttraumatic stress disorder (*n* = 2). Seven patients (41%) already received outpatient psychotherapy and/or psychiatric follow-up.

#### Treatment Recommendations

Psychotherapeutic treatment options were discussed with seven additional patients. Self-help strategies alone were discussed with three patients with rare events who had shown a good understanding of the diagnosis and confidence in the application of self-help strategies to avoid and/or interrupt seizures. Seven of the ten patients (70%) who were not already receiving outpatient psychotherapy and/or psychiatric treatment agreed to follow-up through phone and/or e-mail 1 month after discharge. Two patients did not agree to follow-up as they found it difficult to accept the diagnosis of PNES while one other patient assured that he would reach out in case of questions or new challenges but did not want to be contacted by the hospital.

#### Follow-Up

All seven patients who had agreed to follow-up could be reached through phone or e-mail; all of these patients were adhering to the therapeutic recommendations, i.e., two patients were practicing self-help strategies and five patients had requested to be put on a psychotherapist’s waiting list.

### Patients With Non-epileptic Events Other Than Psychogenic Non-epileptic Seizures

Screening followed by a standardized interview revealed a psychiatric diagnosis in seven of 16 patients (44%) with a diagnosis of non-epileptic events other than PNES (see Figure 1). Four of them were women and their median age was 49 years (range: 19–65 years). Two of them were diagnosed with both a depressive episode and an anxiety disorder, two patients had a depressive episode, two had an adjustment disorder, and one patient was diagnosed with a substance use disorder. Three of these seven patients had not been diagnosed with a psychiatric disorder previously.

## Discussion

This naturalistic study investigated outcomes of a recently established psychotherapeutic service that included routine screening with validated screening measures, a psychotherapeutic session with positively screened patients and PNES patients as well as the development of a personalized treatment plan enhanced by the distribution of supportive resources and a systematic follow-up to evaluate adherence to therapeutic recommendations. In line with previous studies more epilepsy patients with an anxiety disorder had previously been undiagnosed compared to those with a depressive episode (63% vs. 30%), and the majority of patients with a psychiatric diagnosis was not receiving any mental health care at the time of admission, including patients in whom a psychiatric diagnosis had previously been established (Scott et al., 2021). While other routine screening procedures tend to focus solely on epilepsy patients (Metternich et al., 2012; Brandt et al., 2014), an early and inclusive screening procedure was applied in this naturalistic study by including patients with hitherto unclassified events and probable non-epileptic events other than PNES. A broad spectrum of psychiatric disorders including previously undiagnosed adjustment disorder was established in this subsample warranting psychological treatment. This finding may reflect the often overlooked burden of uncertainty in patients who endure long waiting times to undergo specialized diagnostic procedures in tertiary care settings.

While a wealth of epidemiological studies has repeatedly confirmed the high rates and clinical significance of undertreated psychiatric comorbidities in epilepsy (Mula et al., 2021), clinical studies that investigate the outcomes of actually implemented screenings and treatment recommendations informed by these epidemiological findings are unfortunately scarce (Gandy et al., 2021). Thus, this naturalistic study is one of the few studies that provide pragmatic guidance on the integration of psychological care within epilepsy care including the formulation of treatment recommendations and systematic follow-up to evaluate outpatient adherence (Ward et al., 2020). Considering patients’ preferences, treatment recommendations were discussed with the majority of patients who were not already receiving any treatment and follow-up yielded high adherence rates in epilepsy and PNES patients (94 and 100%). In comparison to other studies the initial adherence to therapeutic recommendations in this study is high, particularly in PNES patients (Goldstein et al., 2020; Stone et al., 2020). Previous studies have demonstrated the clinical relevance of strengthening patient motivation when discussing therapeutic recommendations with PNES patients (Tolchin et al., 2019). We assume that our formulation and communication of a personalized psychological treatment plan effectively addressed patient ambivalence and thus strengthened patient motivation to adhere to therapeutic recommendations.

### Limitations

This study has several limitations inherent to its naturalistic design. So far, our psychotherapeutic service included the systematic screening of patients with an admission appointment. Thus, patients who were admitted through the emergency ward have not been included systematically and outpatients have not been included at all. The possibility to extend our psychotherapeutic service is limited by personnel resources, as both psychotherapists work part-time and are externally funded. While professional societies recommend the integration of trained mental health staff in neurology, insurance reimbursement schemes do not necessarily allow for such transdisciplinary treatment.

In regard to our screening procedures, the NDDI-E was used to screen patients with unclassified events for depression even though it has only been validated in epilepsy patients. As the sensitivity of the NDDI-E in patients with non-epileptic events is not known, this may have led to a higher rate of undetected depression in these patients than in an epilepsy sample. As a positive screening result was followed by a standardized interview for mental health disorders as the gold standard of confirming psychiatric diagnoses, the unknown specificity for this subsample was irrelevant in this study.

In addition, our follow-up period was very short and the follow-up information about adherence to therapeutic recommendations was only obtained through patient self-report without additional verification. Furthermore, our follow-up focused on the discussion of potential challenges with the adherence to therapeutic recommendations and we have not systematically obtained other self-reported measures to assess the effect of recommended treatments such as a comparison between adherent and non-adherent patients in regard to depressive or anxiety symptom scores, health-related quality of life, or seizure frequency.

### Future Studies

Much longer follow-up periods than 1 month are needed in order to assess changes that occur in the context of psychotherapy. Additional follow-up data beyond 1 month is currently being collected and will be analyzed in future studies. Future studies should also include additional outcome measures in order to evaluate not only changes in psychiatric symptoms and quality of life but also in medical outcomes. Even though psychiatric comorbidity has been identified as a poor prognostic marker of medical outcomes, prospective studies that show an improvement of medical outcomes in the context of integrated mental health care are lacking. Such studies could also investigate the effects of a nuanced consideration of psychiatric symptoms in regard to ASD choice and titration speed.

## Conclusion

Integrating mental health specialists and establishing standardized psychiatric screening and follow-up procedures improve adherence to mental health care recommendations in epilepsy and PNES patients.

## Data Availability Statement

The raw data supporting the conclusions of this article will be made available by the authors, without undue reservation.

## Ethics Statement

The study involving human participants was reviewed and approved by the Ethics Board of the Ruhr University Bochum (20-7127).

## Author Contributions

RM, SSch, and SP formulated the initial study idea. RM performed data preparation and data analysis. All authors contributed to the implementation of the study, advised on evaluation and reviewed the final manuscript.

## Conflict of Interest

RM receives royalties for German treatment workbooks for seizure patients from Hippocampus and Pabst publishers. The remaining authors declare that the research was conducted in the absence of any commercial or financial relationships that could be construed as a potential conflict of interest.

## Publisher’s Note

All claims expressed in this article are solely those of the authors and do not necessarily represent those of their affiliated organizations, or those of the publisher, the editors and the reviewers. Any product that may be evaluated in this article, or claim that may be made by its manufacturer, is not guaranteed or endorsed by the publisher.

## References

[B1] Brandt C. Labudda K. Illies D. Schöndienst M. May T. W. (2014). Schnelle erkennung einer depressiven störung bei menschen mit epilepsie. *Der Nervenarzt* 85 1151–1155. 10.1007/s00115-013-3982-6 24463650

[B2] Friedman D. E. Kung D. H. Laowattana S. Kass J. S. Hrachovy R. A. Levin H. S. (2009). Identifying depression in epilepsy in a busy clinical setting is enhanced with systematic screening. *Seizure* 18 429–433. 10.1016/j.seizure.2009.03.001 19409813

[B3] Gandy M. Modi A. C. Wagner J. L. LaFrance W. C. Jr. Reuber M. Tang V. (2021). Managing depression and anxiety in people with epilepsy: a survey of epilepsy health professionals by the ILAE psychology task force. *Epilepsia Open* 6 127–139. 10.1002/epi4.12455 33681656 PMC7918327

[B4] Gill S. J. Lukmanji S. Fiest K. M. Patten S. B. Wiebe S. Jetté N. (2017). Depression screening tools in persons with epilepsy: a systematic review of validated tools. *Epilepsia* 58 695–705. 10.1111/epi.13651 28064446

[B5] Gilliam F. G. Barry J. J. Hermann B. P. Meador K. J. Vahle V. Kanner A. M. (2006). Rapid detection of major depression in epilepsy: a multicentre study. *Lancet Neurol.* 5 399–405. 10.1016/s1474-4422(06)70415-x 16632310

[B6] Goldstein L. H. Robinson E. J. Mellers J. D. C. Stone J. Carson A. (2020). Cognitive behavioural therapy for adults with dissociative seizures (CODES): a pragmatic, multicentre, randomised controlled trial. *Lancet Psychiatry* 7 491–505. 32445688 10.1016/S2215-0366(20)30128-0 PMC7242906

[B7] Kanner A. M. Barry J. J. Gilliam F. Hermann B. Meador K. J. (2010). Anxiety disorders, subsyndromic depressive episodes, and major depressive episodes: do they differ on their impact on the quality of life of patients with epilepsy? *Epilepsia* 51 1152–1158. 10.1111/j.1528-1167.2010.02582.x 20477847

[B8] Kerr M. P. Mensah S. Besag F. de Toffol B. Ettinger A. Kanemoto K. (2011). International consensus clinical practice statements for the treatment of neuropsychiatric conditions associated with epilepsy. *Epilepsia* 52 2133–2138. 10.1111/j.1528-1167.2011.03276.x 21955156

[B9] LaFrance W. C. Jr. Baker G. A. Duncan R. Goldstein L. H. Reuber M. (2013). Minimum requirements for the diagnosis of psychogenic nonepileptic seizures: a staged approach: a report from the international league against epilepsy nonepileptic seizures task force. *Epilepsia* 54 2005–2018. 10.1111/epi.12356 24111933

[B10] Löwe B. Decker O. Müller S. Brähler E. Schellberg D. Herzog W. (2008). Validation and standardization of the generalized anxiety disorder screener (GAD-7) in the general population. *Med. Care* 46 266–274. 10.1097/mlr.0b013e318160d093 18388841

[B11] Margraf J. Cwik J. C. Pflug V. Schneider S. (2017). Strukturierte klinische Interviews zur erfassung psychischer störungen über die Lebensspanne. *Zeitschrift Klinische Psychol. Psychotherapie* 46 176–186. 10.1026/1616-3443/a000430

[B12] Margraf J. C. (2017). *Mini-DIPS Open Access: Diagnostic Short-Interview for Mental Disorders. [Mini-DIPS Open Access: Diagnostisches Kurzinterview bei psychischen Störungen].* Bochum: R.-U. Forschungs- und Behandlungszentrum für psychische Gesundheit.

[B13] Metternich B. Wagner K. Buschmann F. Anger R. Schulze-Bonhage A. (2012). Validation of a german version of the neurological disorders depression inventory for epilepsy (NDDI-E). *Epilepsy Behav.* 25 485–488. 10.1016/j.yebeh.2012.10.004 23153711

[B14] Michaelis R. Tang V. Goldstein L. H. Reuber M. LaFrance W. C. Jr. (2018). Psychological treatments for adults and children with epilepsy: evidence-based recommendations by the international league against epilepsy psychology task force. *Epilepsia* 59 1282–1302. 10.1111/epi.14444 29917225

[B15] Mula M. Kanner A. M. Jetté N. Sander J. W. (2021). Psychiatric comorbidities in people with epilepsy. *Neurol Clin Pract.* 11 e112–e120. 33842079 10.1212/CPJ.0000000000000874 PMC8032418

[B16] Patel A. D. Baca C. Franklin G. Herman S. T. Hughes I. Meunier L. (2018). Quality improvement in neurology: epilepsy quality measurement set 2017 update. *Neurology.* 91 829–836. 10.1212/WNL.0000000000006425 30282773

[B17] Petrovski S. Szoeke C. E. Jones N. C. Salzberg M. R. Sheffield L. J. Huggins R. M. (2010). Neuropsychiatric symptomatology predicts seizure recurrence in newly treated patients. *Neurology* 75 1015–1021. 10.1212/wnl.0b013e3181f25b16 20837970

[B18] Pfäfflin M. Schmitz B. May T. W. (2016). Efficacy of the epilepsy nurse: results of a randomized controlled study. *Epilepsia* 57 1190–1198. 10.1111/epi.13424 27265887

[B19] Popkirov S. Asadi-Pooya A. A. Duncan R. Gigineishvili D. Hingray C. Miguel Kanner A. (2019). The aetiology of psychogenic non-epileptic seizures: risk factors and comorbidities. *Epileptic Disord.* 21 529–547. 31843732 10.1684/epd.2019.1107

[B20] Popkirov S. Grönheit W. Jungilligens J. Wehner T. Schlegel U. Wellmer J. (2020). Suggestive seizure induction for inpatients with suspected psychogenic nonepileptic seizures. *Epilepsia* 61 1931–1938. 10.1111/epi.16629 32712967

[B21] Scott A. J. Sharpe L. Thayer Z. Miller L. A. Nikpour A. Parratt K. (2021). How frequently is anxiety and depression identified and treated in hospital and community samples of adults with epilepsy? *Epilepsy Behav.* 115:107703. 10.1016/j.yebeh.2020.107703 33423019

[B22] Seo J. G. Cho Y. W. Lee S. J. Lee J. J. Kim J. E. Moon H. J. (2014). Validation of the generalized anxiety disorder-7 in people with epilepsy: a MEPSY study. *Epilepsy Behav.* 35 59–63. 10.1016/j.yebeh.2014.04.005 24798411

[B23] Stone J. Callaghan H. Robinson E. J. Carson A. Reuber M. Chalder T. (2020). Predicting first attendance at psychiatry appointments in patients with dissociative seizures. *Seizure* 74 93–98. 10.1016/j.seizure.2019.11.014 31869756

[B24] Tolchin B. Baslet G. Suzuki J. Martino S. Blumenfeld H. Hirsch L. J. (2019). Randomized controlled trial of motivational interviewing for psychogenic nonepileptic seizures. *Epilepsia* 60 986–995. 10.1111/epi.14728 30980679

[B25] Walther K. Volbers B. Erdmann L. Dogan Onugoren M. Gollwitzer S. Kasper B. S. (2019). Psychological long-term outcome in patients with psychogenic nonepileptic seizures. *Epilepsia* 60 669–678. 30838655 10.1111/epi.14682

[B26] Wang Z. Luo Z. Li S. Luo Z. Wang Z. (2019). Anxiety screening tools in people with epilepsy: a systematic review of validated tools. *Epilepsy Behav.* 99:106392. 10.1016/j.yebeh.2019.06.035 31521915

[B27] Ward W. L. Smith A. Munns C. Bai S. (2020). The process of integrating psychology into medical clinics: pediatric psychology as an example. *Clin. Child Psychol. Psychiatry* 26 323–341. 10.1177/1359104520982323 33353382

